# A New Configurable Wireless Sensor System for Biomedical Applications with ISO 18000-3 Interface in 0.35 µm CMOS †

**DOI:** 10.3390/s19194110

**Published:** 2019-09-23

**Authors:** Tatjana Fedtschenko, Alexander Utz, Alexander Stanitzki, Andreas Hennig, Andre Lüdecke, Norbert Haas, Rainer Kokozinski

**Affiliations:** 1 Fraunhofer IMS, 47057 Duisburg, Germany; Alexander.Utz@ims.fraunhofer.de (A.U.); Alexander.Stanitzki@ims.fraunhofer.de (A.S.); Andreas.Hennig@ims.fraunhofer.de (A.H.); Andre.Luedecke@ims.fraunhofer.de (A.L.);; 2 Department of Electronic Components and Circuits, University of Duisburg-Essen, 47057 Duisburg, Germany

**Keywords:** biosensor, potentiostat, amperometry, wireless, RFID, NFC, 13.56 MHz, ISO 18000-3

## Abstract

This article presents a new configurable wireless sensor system. The system is used to perform amperometric measurements and send the measurement data to a handheld reader using a wireless transponder interface. The two-chip sensor system was implemented in a 0.35 μm CMOS technology. The system consists of an integrated nano-potentiostat that performs the actual measurements and an ISO 18000-3 compliant frontend that enables wireless telemetric data transmission and powering of the entire sensor system. The system was manufactured in combination with a chronoamperometric glucose sensor which allows the measurement of the glucose content in tear fluid and thus a non-invasive determination of the blood sugar level. For a range of sensor currents from 0.1 μA to 10 μA, the potentiostat achieved an accuracy of better than 5 % with a total power dissipation of less than 600 μW. With the realized antenna geometry a wireless communication distance of more than 7 cm has been achieved.

## 1. Introduction

For a wide range of applications, such as environmental measurement, process control, and biomedical monitoring, electrochemical sensors converting the chemical concentration into an electrical signal are used. Examples include applications such as in vivo temperature measurement, lactate concentration measurements, or determination of pH and glucose levels [1,2,3,4].

The combination of sensor and sensing electronics enables to get miniature systems with built-in signal processing and configuration capabilities. Additional integration of the analog-to-digital converter allows wireless transmission of sensor data using standardized digital protocols.

This article presents a passive biochemical microsystem consisting of two microchips: A biochemical sensor interface (potentiostat) and a chip for wireless energy and data transfer based on the ISO18000-3 protocol [5] (transponder).

The nano-potentiostat regulates a 3-electrode system with enzyme electrodes. Different enzymes can analyze not only glucose but also other molecules, e.g., Lactate, Xanthan, Ethanol. The measurement of the lactate content in human blood is of interest for athletes in order to optimally shape longer training sessions and competition preparations. The food industry is interested in the determination of Xanthan concentrations as these are indicators of the freshness of fish and meat products.

The composite architecture was chosen to ensure the reuse of the developed communication interface in other biomedical applications and to provide the possibility of combining several measuring sensors with one communication interface [6] which makes the system expandable and configurable. The biochemical sensor interface (potentiostat) presented in this article includes an amperometric measuring circuit and, a digital part including an EEPROM, and a SPI slave interface. The transponder consists of an antenna, a high-frequency (HF) frontend, a digital base band, and a standardized master/slave SPI interface. This microsystem demonstrates the ability to miniaturize glucose measuring tool. It can be used as a non-invasive glucometer for continuous monitoring of glucose levels in the loop for continuous health monitoring.

**Figure 1:**
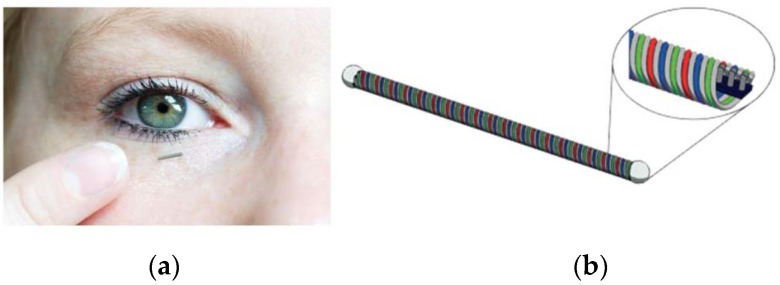
Concept of the wireless glucose sensor [7]: (**a**) Application; (**b**) flexible coil shaped electrodes.

The developed miniaturized amperometric system was tested on a device that determines glucose concentration by directly measuring of "normal" or basal tear fluid production. [7]. The measuring system was realized as a coil shaped device. A coil represents a device with high flexibility perpendicular to the short axis which can adapt to the shape of an eye and at the same time is an ideal device to act as an antenna for wireless data and power supply (Figure 1b). To measure glucose levels amperometrically, three electrodes are wound in parallel to form an amperometric cell, while a fourth wire is used to facilitate wireless data and energy transfer. Figure 1a shows the concept of the wireless glucose sensor that can be worn under the eyelid [7].

## 2. System Architecture

**Figure 2:**
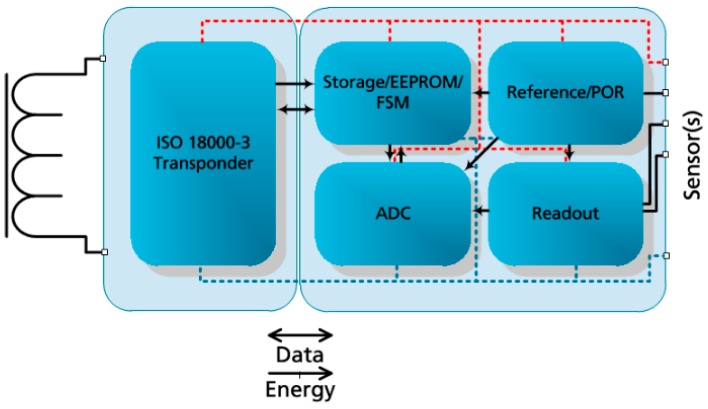
Telemetric sensor system architecture.

Figure 2 shows the architecture of the portable sensor microsystem, comprising a transponder ASIC (application specific integrated circuit) and a potentiostat ASIC including readout, analog to digital conversion (ADC), reference and power-on-reset (POR) generation blocks as well as storage and control block with connected electrodes [7].

The energy is transmitted wirelessly from the reader to the transponder ASIC. The supply voltage for the entire system is generated by a voltage rectifier in the transponder. The transponder also decodes incoming commands from the reader unit. Further it encodes and transmits the processed measurement results back to the reader where they are stored and can be subjected to further processing.

The main function of the potentiostat is to measure current from the connected electrodes. In addition, the chip contains an interface for data exchange, a temperature sensor, a voltage control sensor, and an EEPROM for integrated linearization and calibration data storage. The current is measured from the sensor using a current to voltage converter composed of a low noise transimpedance amplifier (TIA) followed by analog-to-digital converter. Unlike analogue direct current to frequency conversion approaches [1,8] this implementation provides compatibility with NFC and RFID standards based on ASK load modulation for wireless data transmission as well as the capability to transmit identification and calibration data.

Built-in calibration data storage and dual-chip architecture provide a very flexible sensor system that can be easily adapted to various applications.

## 3. Circuit Implementation: ISO 18000-3 RF Frontend (Transponder)

**Figure 3:**
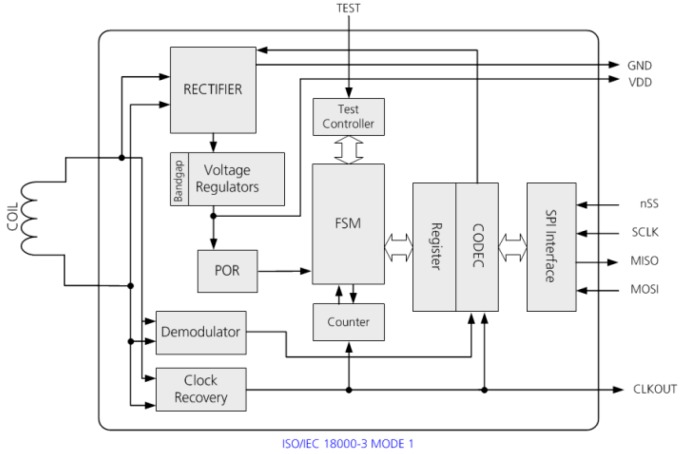
The transponder block diagram.

The analog section of the RF interface (transponder) uses as an interface for wireless communication a carrier frequency of 13.56 MHz (ISO/IEC 18000-3). Figure 3 shows a block diagram of this section.

In addition to encoding-decoding and communication with the reader, the transponder powered by the RF field coordinates the actions of the potentiostat via a standard compatible serial peripheral interface (SPI). Sensor values and other digital data, such as status information from a potentiostat, are transmitted to the reader by modulating the RF field. The transponder includes the following blocks [6]:

- Voltage rectifier with load modulator and limiting circuitry;
- Voltage regulator with bandgap reference;
- POR;
- Demodulator and clock recovery circuit;
- SPI interface.

### 3.1. Power Delivery

The induced alternating voltage at the antenna coil can vary from 100 mV to more than 20 V depending on the orientation and distance to the reader coil. Thus, during measurement the voltage can fluctuate significantly. Therefore, the remaining modules must be protected against overvoltage and a stable supply voltage needs to be ensured. Hence, voltage limitation and voltage regulation are necessary.

**Figure 4:**
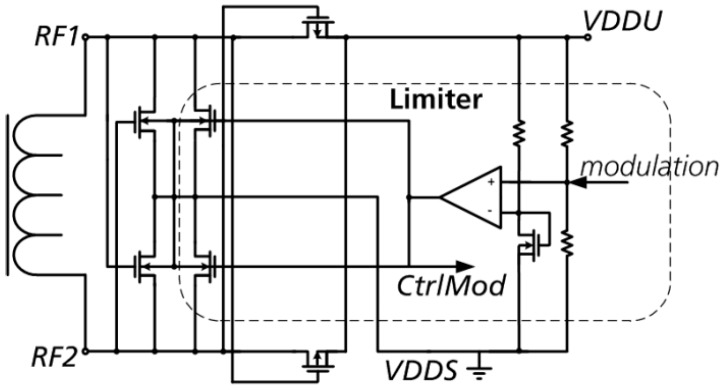
Simplified rectifier circuit with limiter.

The voltage limiter is implemented as an active control loop. As a simple voltage reference a voltage drop across the diode-connected transistor is used. This voltage is compared with the scaled rectifier output voltage by an error amplifier. The error amplifier drives the gate of an NMOS-type limiting device to extract additional current from the rectifier (Figure 4).

To reduce fluctuations in the supply voltage a capacitor-free CMOS low dropout regulator (LDO) is utilized [9]. A temperature independent bandgap voltage acts as reference. In general, the regulated voltage shows a temperature coefficient of −200 ppm/K [6]. Two voltage regulators are used to separate the noise of digital switching from the analog signal processing part: one for analog units (2.6 V) and one for digital circuits (1.8 V).

### 3.2. POR Generation

To avoid an undefined circuit behavior at supply voltages below the specified operating range a power-on-reset (POR) is used that is activated at the lowest possible operating voltages, triggering a global reset, and holding it until the supply voltage reaches a value within the voltage operating range.

The POR is carried out as a time delay. When the supply voltage exceeds the value of 1.25 V, the startup circuit will trigger a switch and the integrated capacity starts charging with current from the bandgap. As soon as the voltage on the capacitance exceeds the lower threshold value, the voltage regulators will be turned on. After the regulated voltage exceeds the upper threshold value, the global reset will be activate. The capacitor charging time determines the duration of the reset pulse.

### 3.3. Demodulator Circuit

**Figure 5:**
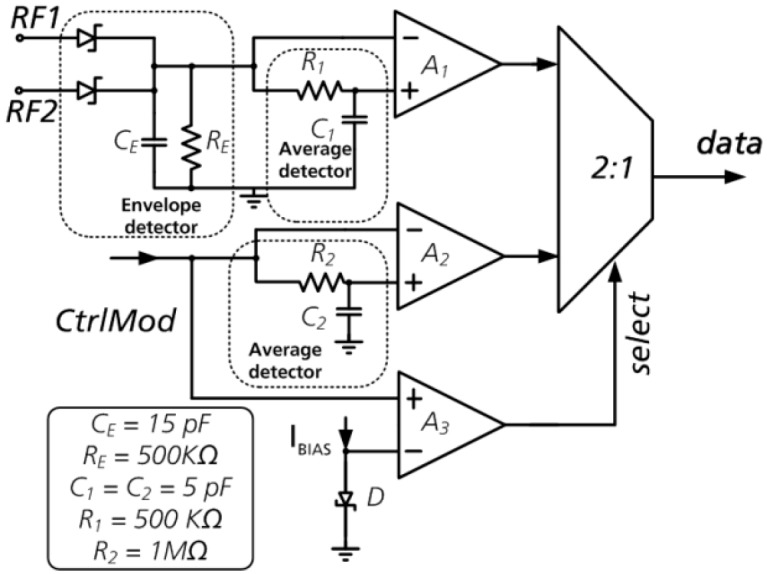
Demodulator with the switch for near and far field.

The demodulator is used to recover 13.56 MHz modulated carrier data in accordance with ISO/IEC 18000-3 MODE 1 [5]. To handle the wide dynamic range a two-stage demodulator was used (Figure 5). If the transponder is located far from the reader, the demodulator will determine the envelope of the carrier emitted by the reader from the antenna input nodes and the hysteresis comparator *A1* will compare this envelope with the average of this envelope and detect transitions between modulated and non-modulated states and vice versa. To approximate the average value a passive low-pass filter is used. In the near field, where the antenna power becomes too large, the limiting circuit is activated. Part of the antenna power is shorted and the antenna amplitude is saturated. In this case the information given by the ASK modulation can be recovered from the voltage control signal *CtrlMod* of the limiter. The envelope and the average value are obtained from this control signal and the incoming modulation is detected by the hysteresis comparator *A2* (the outgoing modulation is disabled at this time). The selection occurs by switching the output of the comparator *A3* which compares the control signal level with the reference voltage. The reference voltage is realized as a voltage drop across the properly biased Zener diode *D*.

### 3.4. Digital Interface

The digital part [6] of the transponder ASIC handles the protocol execution and the communication with the external sensor system. The minimum set of mandatory commands defined in ISO 18000-3 MODE 1 (respectively ISO/IEC 15693) [5] has been implemented to ensure compliance with standard readers. This set consists of the “inventory” command for collision management and the “stay quiet” command to disable responses of a tag. Additionally, the “read-” and “write-single-block” commands have been implemented. The block length is 16 bit. The communication interface with the sensor system is provided by a serial peripheral interface (SPI) with a register width of 16 bit. Block address 0 of the ISO 18000-3 protocol is directly mapped to the SPI shift-register, i.e., the transponder implements a transparent SPI bridge. When a “write-single-block” command with target address 0 is issued, the 16 bit contents of the data field from the ISO protocol frame will be copied to the SPI shift-register followed by a 16 bit shift operation to the connected SPI-slave. When a “read” command is issued, the actual contents of the SPI shift-register will be transmitted via the resulting tag response. Using this method, arbitrary sensors that are accessible over an SPI-interface can be handled.

Timing characterization for a set of digital standard cells available in the target 0.35 µm CMOS process was performed to allow operation at low supply voltages (1.8 V in this case). The protocol execution runs at a system clock of 3.39 MHz which is the minimum level that is required to generate the subcarrier for the tag reply modulation. The major part of the internal state machine and interface runs at a significantly lower speed of 105 kHz that is generated internally. Clock gating was used to further reduce power consumption. This results in average power dissipation of 180 µW for the logic section.

## 4. Circuit Implementation: Sensor Frontend (Potentiostat)

**Figure 6:**
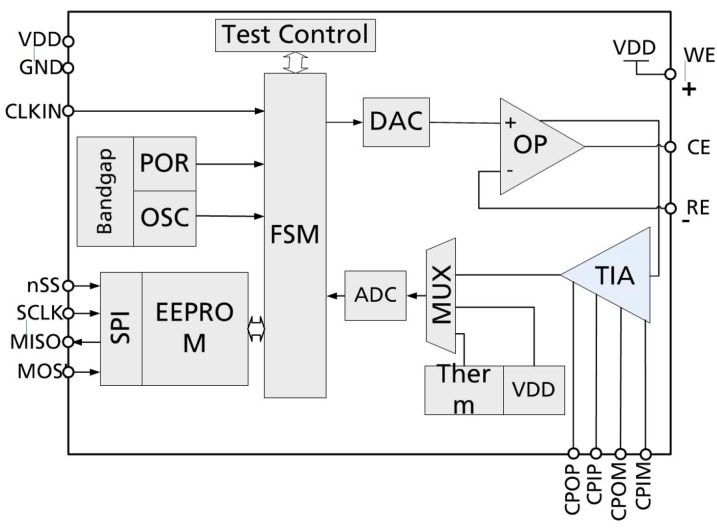
The block diagram of the potentiostat ASIC [6].

The second chip implements the sensor interface (Figure 6). It performs measurements and digital conversion including calibration and linearization of the sensor signal. It contains the following components [6]:

- A temperature sensor and a supply voltage measuring circuit;
- A cyclic ADC with 12 bit resolution and low power consumption;
- A digital to analog converter (DAC) with 6 bit resolution;
- A precision operational amplifier as control for the excitation voltage;
- A TIA for converting the current between the work electrode (WE) and the counter electrode (CE) into a corresponding voltage;
- A state machine for the ADC and measurement sequence timing;
- Dedicated registers for storage and transfer of measurement results;
- A serial EEPROM for storing chip ID, sensor offset/gain and simple calibration constants, e.g., for setting the control voltage and gain at the transimpedance amplifier;
- A charge pump for in-circuit programming of the EEPROM;
- An SPI slave interface for communication with the RF frontend;
- A POR circuit.

Temperature changes affect the steady-state current measured by amperometry. Since the glucose sensor target temperature range is 10 °C–45 °C the current range must be assumed to vary between 100 nA and 3 µA [10]. Furthermore temperature values are transmitted to the reader unit to enable a correction of this effect.

### 4.1. Control Loop: Amplifier and DAC

Performing in the sensor frontend amperometric measurements the voltages are applied to the sensor electrodes causing them to adjust, depending on the solution concentration, electrolysis current. Thus, the measured signal is proportional to the chemical concentration.

The nano-potentiostat ASIC is composed of several parts. The most important element of the ASIC is the potentiostat control circuit which provides the necessary constant bias potential between electrodes WE and CE. The inverting input of the precision rail-to-rail operational amplifier is connected to the terminal for the reference electrode (RE). This allows control of the cell voltage between the working electrode (WE) and RE with the reference voltage set via the DAC. The output of the operational amplifier controls the gate of a PMOS transistor (T1) connected as a source follower and thus controls the current flow through the WE and the counter electrode (CE). The potentiostat thus performs a non-disturbing measurement of the redox current [8].

The reference voltage V_DAC_ applied to the non-inverting input of the operational amplifier is provided by a digital-to-analog converter (DAC). The 6 bit resolution C2C DAC can be used to set reference voltages in the range from 1.4 V up to the supply voltage of 2.6 V in steps of 22 mV. The ADC input value is retrieved from the EEPROM in the initialization.

**Figure 7:**
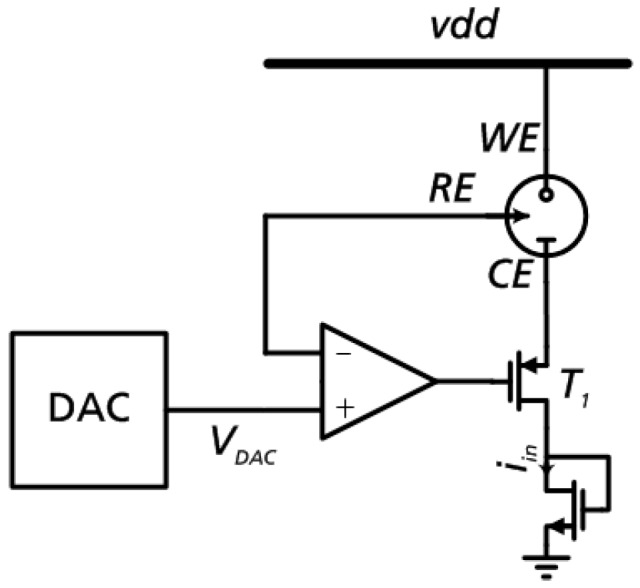
Basic structure of the potentiostat [6].

The block diagram of the system is shown in Figure 7. The polarity of the WE-CE path can be changed depending on the application.

The reference voltage V_DAC_ applied to the non-inverting input of the operational amplifier is provided by a 6-bit DAC. The desired reference potential is stored in the EEPROM as a 6-bit value. The reference voltages ranging from 0 V (DAC value 0) to 1.4 V (DAC value 63) can be set with the DAC with a resolution of 22 mV. The necessary clock for the C2C DAC frequency is generated in the digital part. To reduce interfering peaks on the DAC output reference voltage V_DAC_ is filtered with an RC low-pass filter.

The inverting input of the operational amplifier is connected to the terminal for the reference electrode (RE). This allows control of the cell voltage between WE and RE with V_DAC_ as the reference variable. The output of the operational amplifier controls the gate of a PMOS transistor (T1) and thus regulates the current flow through the WE and the counter electrode (CE).

**Figure 8:**
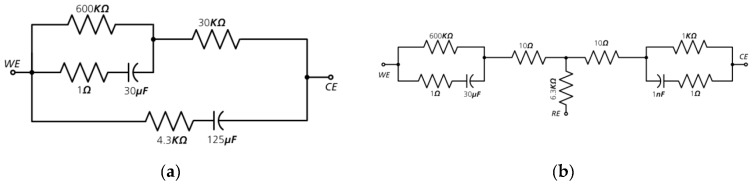
Sensor equivalent circuits: (**a**) 2-electrode sensor; (**b**) 3-electrode sensor [8].

Therefore, an equivalent circuit was used to model the electrical behavior of our glucose sensor in the test bench. The circuit used to emulate the 2- and 3-electrode glucose sensor is shown in Figure 8. The derivation of the values for the sensor equivalent circuit diagram is described in the Appendix A.

The precision rail-to-rail operational amplifier has 60 dB open-loop gain with a maximum power consumption of 220 µW. The phase margin of the sensor control loop determined in the simulation amounts to a minimum of 45°.

### 4.2. Switched-Capacitor TIA

**Figure 9:**
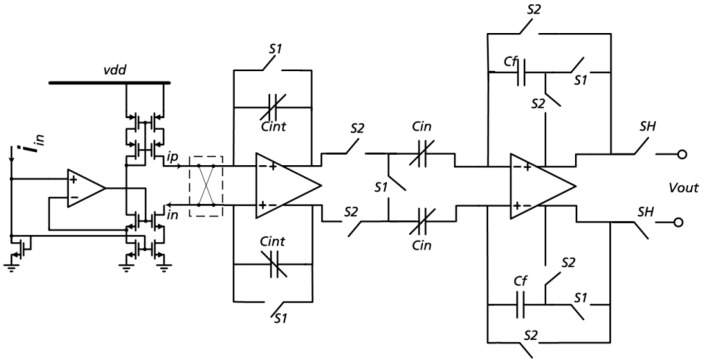
Switched capacitor TIA with correlated double sampling [6].

The current I_in_ flowing from WE to CE results from the applied voltage and the resulting chemical reaction. It is mirrored by single to differential current convertor and measured indirectly using a transimpedance amplifier (TIA, Figure 9) [6]. Two mirrored output currents from the potential control amplifier are used for integration to voltage. This voltage is converted by ADC and stored as a measurement result in one of the result registers.

The potentiostat adapts a current mirror architecture and a low-speed input amplifier to reduce the consumption [8].

The TIA consists of three parts: a single-to-differential input current mirror [11], the actual TIA, and a fully differential switched capacitor sample-and-hold output stage [12]. In the present project the TIA was implemented as a time-controlled switched-capacitor integrator with correlated double sampling (CDS).

The TIA integrates the mirrored output current over a programmable number of cycles giving an adjustable integration time (TI). Start and stop signals for integration are generated by the digital part. During an integration cycle the integration capacity is first loaded with the mirrored current. After an internal switchover the capacity is discharged via the mirrored current with another direction. The result is an output voltage proportional to the difference of the input currents (and thereby to the current in the sensor element).

The number of integration cycles and therefore the integration time is set by a 10-bit calibration word that is retrieved from the EEPROM. The maximum integration time thus is 1000 µs. The TIA has a power consumption of less than 100 µW.

### 4.3. Switched-Capacitor Cyclic ADC

The 12-bit cyclic ADC used in the read-out circuit features has a sample rate of 1 kHz with an input signal range from 0 V to 2.6 V. It is realized in a switched capacitor (SC) technique and implements the RSD scheme that permits to convert an analog input signal within 11 cycles into a 12 bit digital result [12,13,14].

The sequence control of the ADC is clocked via the clock signal coming from the transponder chip. The sequence control carries out exactly one measurement, depending on the set configuration, either glucose content measurement, temperature measurement or voltage measurement.

For each measurement the ADC is reset and then the corresponding signal is sampled by the ADC and a conversion is performed. The conversion result is stored in the associated register at the end of the conversion. After the last conversion the ADC is switched off (powerdown). The on-chip ADC achieved an effective resolution of 12 bit with a power consumption of 75 µW.

### 4.4. References and Auxiliary Sensors

In order to adjust the measurement results to the change in system behavior due to temperature and transmitted energy variations, temperature and supply voltage sensors have been implemented. The linearity error of the temperature sensor with the on-chip bandgap reference is less than ±0.1 K in the measurement range of 10 °C to 45 °C. The integrated power-on-reset (POR) and brown-out detector (BOD) prevent an undefined behavior of the circuit at supply voltages below the specified operating range.

### 4.5. Digital Part

The digital part of the potentiostat is responsible for the execution of the startup procedure of the sensor system, the handling of configuration and calibration data, and EEPROM access control. It also includes the SPI communication interface and controls the execution of single measurements and measurement sequences. Internal 16-bit registers are provided to store the measurement data until they are retrieved by the transponder and sent back to the controlling reader unit. The EEPROM is in-circuit programmable so that a fully assembled sensor device can be calibrated and calibration results can be stored in the device.

The digital control logic runs at a supply voltage of 2.6 V at a system clock of 105 kHz. Clock gating of idle segments is performed to further reduce current consumption. The resulting average power dissipation is approx. 50 µW.

## 5. Results

**Figure 10:**
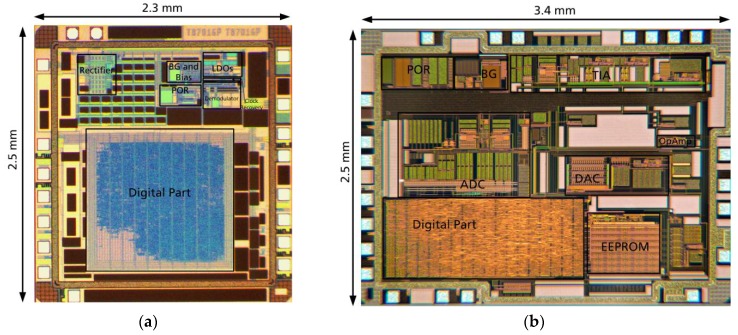
ASICs microphotographs: (**a**) Transponder ASIC; (**b**) potentiostat ASIC.

Both ASICs are manufactured in Fraunhofer IMS 0.35 μm CMOS technology. Figure 10 illustrates micrographs of the system ASICs.

Before characterizing the entire system, validating simulations were performed. To characterize the system the measurements were performed for each chip individually as well as for the entire system. The communication functions of the transponder were tested with the ISO 18000-3 compliant reader. The distance between the antenna and the read coils was varied. The measurement results show that the transponder works safely at a distance of up to 7 cm.

### 5.1. Characteriszation of the RF Frontend

**Figure 11:**
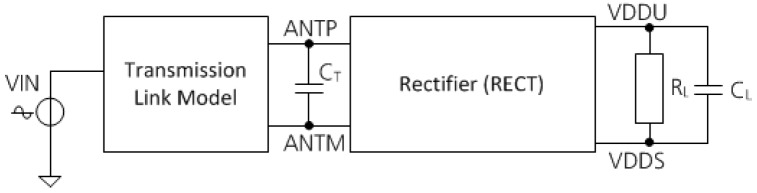
Rectifier simulation test setup.

The rectifier was also simulated in combination with a model of the transmission link as well as a tuning capacity C_T_ and a load resistance R_L_. The simulation test setup is shown in Figure 11.

In this test measurement the RF Frontend input was driven with the signal generator. The measurements on the unregulated output voltage were performed using a Rohde and Schwarz HMO 1002 oscilloscope.

To stabilize the unregulated rectifier output voltage VDDU a buffer capacitor is integrated. A connection pad is led out so that an external buffer capacitor can be connected as well.

**Figure 12:**
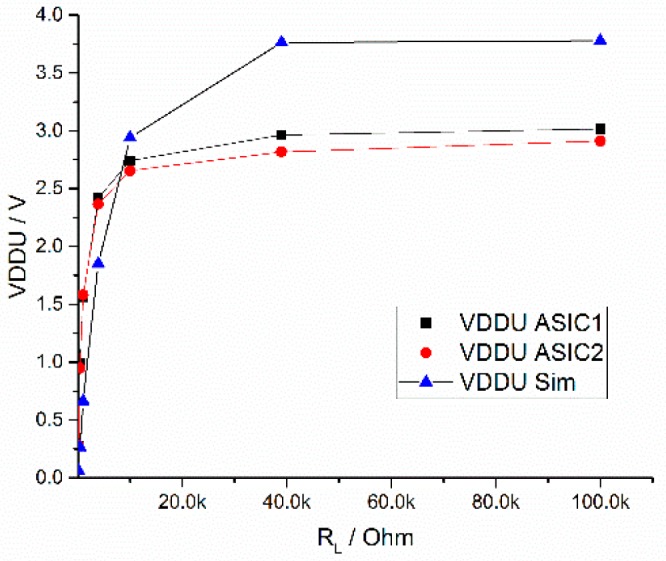
Simulated and measured rectifier output voltage VDDU vs. R_L_ at 13.56 MHz.

Figure 12 shows the dependence of the unregulated output voltage on the resistive load RL in the range of 100 Ω to 100 kΩ. A sinusoidal input voltage was used with a fixed amplitude of 4.5 V and a frequency of 13.56 MHz was used in this measurement. During the measurements, a capacitance of 10 nF was added parallel to the RL to smooth the residual ripple.

With an AC input amplitude of 4.5 V, the DC output voltage reaches 3 V at a load greater 10 kOhm. The rectifier can supply an additional load of down to 15 kOhm. Therefore the output voltage is 2.6 V at minimum, which is sufficient for the power supply of the potentiostat circuit.

### 5.2. Characteriszation of the Potentiostat

#### 5.2.1. Measurements with a Resistance Decade Box to Characterize the TIA

**Figure 13:**
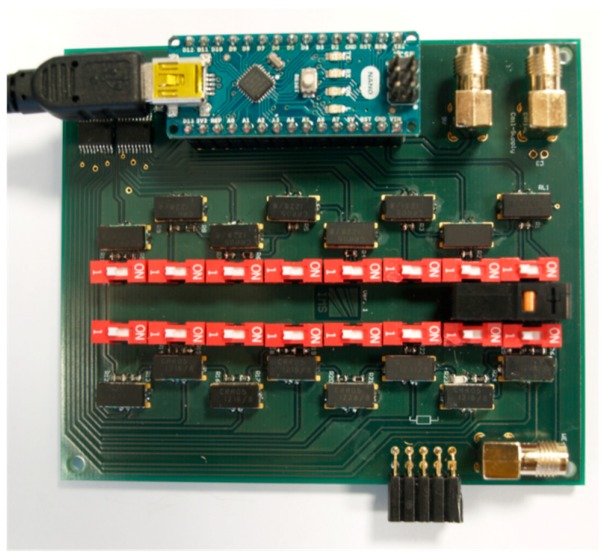
Assembled SMD resistor decade box.

The first measurements to characterize the TIA were carried out with a resistance decade box to produce defined impedances and allow performing reproducible measurements. In order to combine the variability of a resistor decade with the lower noise of SMD resistors, a programmable SMD resistor decade box was designed and developed as a PCB layout (Figure 13). The SMD resistor decade box is realized as binary weighting SMD resistors that add up to a total resistance in a range of 16 bits with a resolution of 1 kOhm. The automation is realized by means of an embedded in an *Arduino nano* µ-controller. The µ-controller was programmed using the *Arduino 1.6.11* development environment using libraries for SPI communication. The µ-controller was connected to the PC via a serial interface.

The “resistance decade” was used to characterize the current driving capability of the potentiostat circuit and the linearity of the transimpedance amplifier.

To measure the functionality and accuracy of the TIA the automatable SMD resistor decade used as variable resistance values is switched between WE and CE at different cell voltages. During this measurements the electrodes RE and CE remained connected together. As mentioned in Section 4.2 the integration time of the TIA can be set as a 10-bit value. If the integration time is known, the detected ADC value change at constant cell voltage for changed resistance values was used to calculate the current I_SENSOR_.

(1) $$I_{\mathrm{SENSOR}}=-K_{\mathrm{gain}}\left(D_{\mathrm{out},I}*U_{\mathrm{LSB}}-V_{\mathrm{CM}}\right)*\frac{C_{\mathrm{int}}}{TI}+K_{\mathrm{off}}$$

where $I_{\mathrm{SENSOR}}$ is the sensor current, $K_{\mathrm{gain}}$ the voltage- and temperature-dependent amplification coefficient, $D_{\mathrm{out},I}$ the digital output for current measurement, $U_{\mathrm{LSB}}$ the LSB Step size of the ADC, $V_{\mathrm{CM}}$ the start voltage of the integrator, $C_{\mathrm{int}}$ the integration capacity, TI the adjustable integration time and $K_{\mathrm{off}}$ an unknown systematic offset.

Characteristics of the TIA are recorded in the entire cell voltage range of 0–1.2 V. In order to be able to evaluate the measurements statistically a measurement series of 20 measurements with 3 ADC samples per measurement is carried out for each resistance value. From these 60 ADC values the mean value and the standard deviation are calculated.

**Figure 14:**
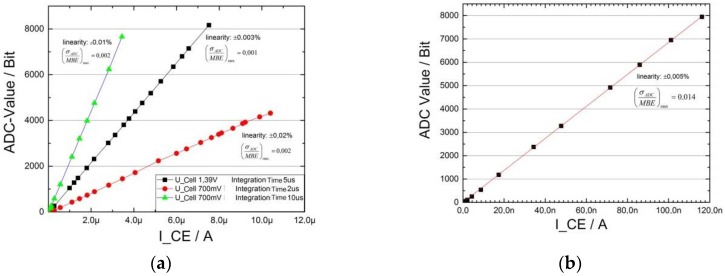
TIA characteristics from the measurement with the “resistor decade”: (**a**) current range about 10 µA; (**b**) current range < 100 nA.

At a current of about 10 μA the deviation from linearity is well below 0.05 % (Figure 14a). When detecting currents I_SENSOR_ < 100 nA large integration times must be used. In this current range the standard deviation is significantly larger (Figure 14b).

#### 5.2.2. Electrochemical Measurements

For the electrochemical measurements the ASIC was connected to screen printed electrodes on a ceramic carrier BST3-WE-Au, CE-Pt, RE − Ag + Ag/AgCl from BST Bio Sensor Technology GmbH. The electrodes were placed in a volume of 20 ml phosphate buffered saline (PBS) solution. To this solution small amounts of 35% H_2_O_2_ solution were added over time. After each addition of 35 % H_2_O_2_ solution a measurement was performed. The cell voltage of 700 mV was chosen.

In laboratory measurements the test setup must be connected to a fixed ground potential. This cause induced electromagnetic interference that affects the RE potential. In order to exclude this disturbance, the measurements were carried out with the shielded and with non-shielded laboratory glass. In the end application the ground potential is floating so that this effect does not occur. Concentration series were carried out for two different integration times.

**Figure 15:**
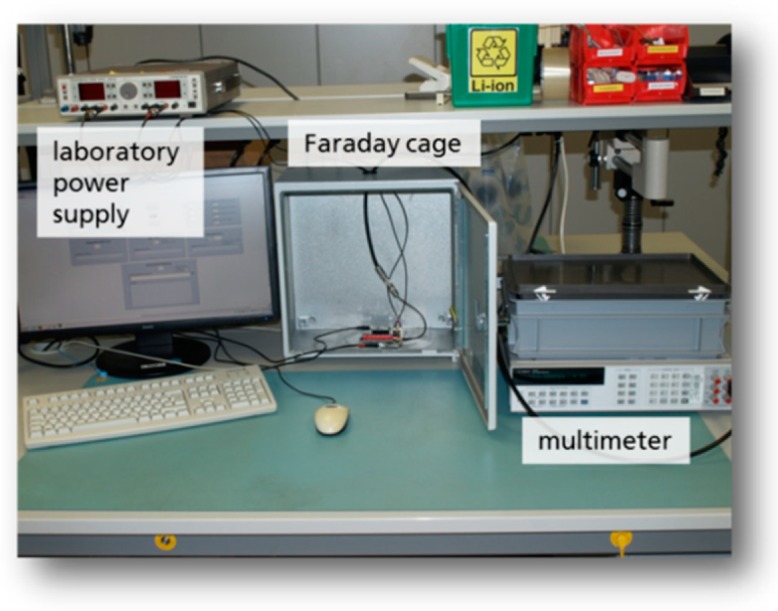
Automated measurement setup with LabView.

In order to carry out automated measurements with the potentiostat ASIC a LabView control software was programmed (Figure 15).

**Figure 16:**
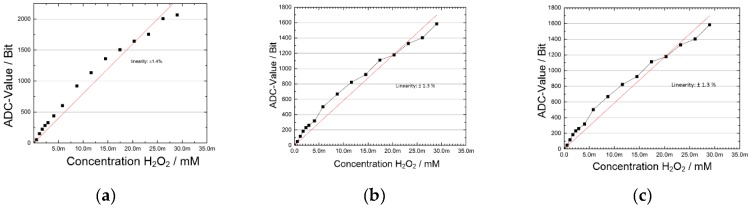
Comparison of H_2_O_2_ concentration measurement with potentiostat ASIC: (**a**) Non-shielded laboratory glass, integration time 5 µs; (**b**) shielded laboratory glass, integration time 5 µs; (**c**) shielded laboratory glass, integration time 25 µs.

The results for several measurement cycles are shown in Figure 16. With an integration time of TI = 5 µs, there is little difference in linearity between the measurements with and without the use of shielding. However, by setting an integration time of TI = 5 µs the linearity became slightly worse as by setting an integration time of TI = 25 µs (Figure 16b,c). The measured ADC resolution is 12 bits, which is equivalent to the resolution of 1 mMol H_2_O_2_ concentration.

**Figure 17:**
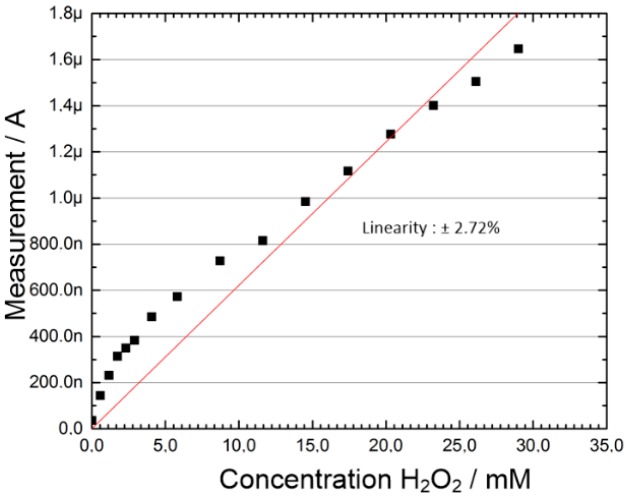
H_2_O_2_ concentration measurement with μAutolabIII.

For reference purposes a commercial lab potentiostat from Metrohm Autolab (µAutolab type III) was used to verify the measured currents. The same type of screen printing electrodes was used. (Figure 17).

In these measurements of the H_2_O_2_ concentration series no statistical evaluation of the measurement results was carried out.

#### 5.2.3. Wireless Concentration Measurement

**Figure 18:**
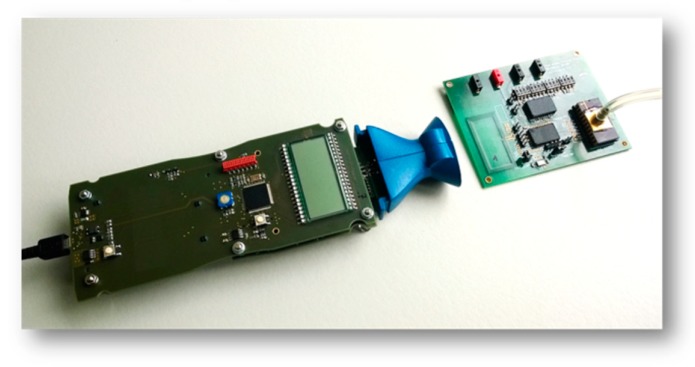
Equipped circuit board with reader device.

**Figure 19:**
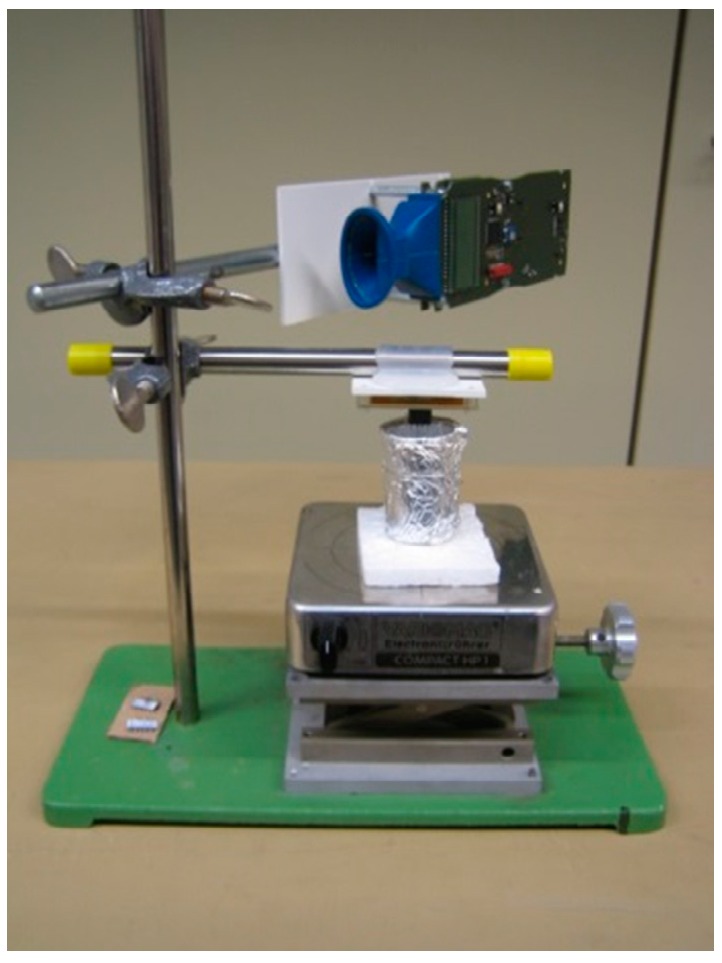
Complete test setup on a laboratory stand [6].

In order to generate reproducible conditions the complete system, i.e., the arrangement of the test board for the potentiostat chips, the transponder chips and the 13.56 MHz reader (Figure 18) were assembled on a laboratory setup (s. Figure 19). The system was measured with sensor electrodes and PCB loop antenna for communication purposes.

A test range was performed in which the concentration of the solution was increased in 5 μL steps to 35 mMol Glucose.

Performing amperometric measurements of a glucose concentration series requires the preparation of an enzyme solution that could be applied to the 3-electrode system. The enzyme solution consists of three components. In PBS, the actual enzyme, the glucose oxidase, is dissolved. The addition of bovine serum albumin (BSA) and glutaldehyde ensures the cross-linking of the enzymes with the BSA and the formation of a matrix. The enzyme solution was dropped on the screen printing electrodes. After the solution has dried, an enzyme layer bound to the ceramic carrier stays behind. The enzyme glucose oxidase cleaves the glucose present in the electrolyte and H_2_O_2_ is produced. When measuring in glucose concentration series, H_2_O_2_ is converted at the working electrode too. The amperometric measurements were therefore also carried out at U_Cell = 700 mV. A concentration series was prepared in 20 ml PBS by adding a 1 mM glucose solution. The physiological range of glucose concentration comprises 3 mM < C_Glucose_ < 30 mM. In this concentration range, the measurements were carried out with the enzyme-coated electrodes. Since the enzyme layer has only a limited lifetime after the electrodes come into contact with the electrolyte, the amperometric measurements were performed as individual measurements. The statistical evaluation of the measurement results has therefore not been carried out.

**Figure 20:**
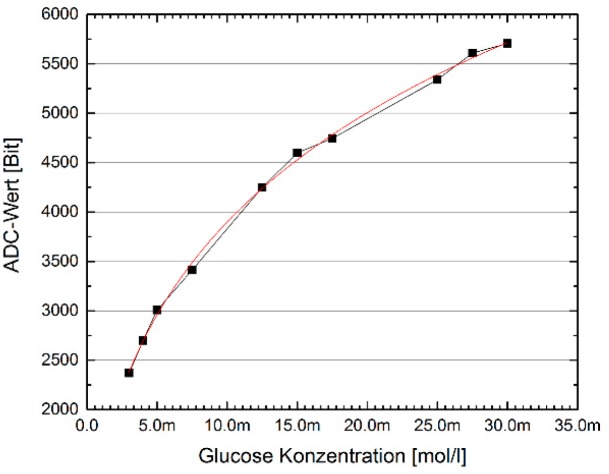
Result of the concentration series.

The diagram (Figure 20) shows that the output values of the ADC in the potentiostat chip behave strictly monotonously increasing with the concentration of the Glucose analyte. The measurementreadings were fitted with the Hill function. The course of the ADC values as a function of the glucose concentration of the electrolyte fulfills the expectations based on the enzyme kinetics.

#### 5.2.4. Experimental Measurements

The sensor system approach was practically evaluated regarding the ability to measure glucose level in the tear fluid. The carrying out of the experimental measurements is described more detailed in [15].

The sensor system was calibrated using a concentration of 0–16 mM glucose. Calibration curves were obtained by gradually increasing the glucose concentration.

The sensor system shows excellent linearity in the concentration range of 0 to 20 mM in the measurement of glucose concentration in tear fluid. A series of in vivo tests were performed to determine the accuracy of the sensor system. The data obtained from animal experiments show that 92% of the collected data fall into regions A and B giving a sufficient accuracy [15]. It has been demonstrated that a non-invasive biosensor can be used to measure glucose in the tear fluid to accurately record glucose levels in a clinical setting.

## 6. Conclusions

In this article we presented a wireless, portable, and flexible RFID sensor microsystem realized as dual-chip system, consisting of glucose sensor, potentiostat circuit, and ISO 18000-3 transponder interface. The key system characteristics are summarized in the Table 1.

The described realization of the glucose measuring system comprises a measuring range of 100 nA–100 μA. Due to parameterizability using an integrated EEPROM the sensitivity/integration time and the measuring range can be set by programming. The adjustable cell voltage can be changed in 64 steps. Supply voltage and temperature integrated sensors allow system self-calibration and conversion of measured current values to equivalent compound concentrations.

The complete demonstrator system including ASIC manufacturing was implemented in 0.35 µm CMOS technology and validated with handheld reader wireless measurements. Finally, the system was tested under clinically relevant conditions.

The measurement results for the H_2_O_2_ and glucose detection allow to assume that with the potentiostat the concentration of the substances Lactate and Xanthan is also determinable, since the detection methods on the enzymatic conversion are very similar.

Table 2 compares our system with the prior devices for wireless amperometric measurements.

## Figures and Tables

**Table 1:** Key System Characteristics.

Parameter	Value
Modulation scheme	ASK
Carrier Frequency	13.56 MHz
Regulated Supply Voltage	2.6 V analog; 1.8 V digital
Power consumption	
Transponder	<300 µW
potentiostat	<300 µW
Full scale measured current	100 µA
Wirelessly readout distance	7 cm
Chip size	
Transponder	5.75 mm^2^
potentiostat	8.5 mm^2^

**Table 2:** Comparison with Previous Works.

Parameter	This Work	[16]	[17]	[18]	[8]	[1]
Carrier Frequency	13.56 MHz	13.56 MHz	13.56 MHz	13.56 MHz	13.56 MHz	1.8 GHz
Protocol	ISO/IEC 18000-3	ISO 15693	ISO 15693	ISO 15693	N/A	N/A
Modulation scheme	2-ASK	LSK	LSK	LSK	LSK	LSK
Glucose detection level	0–20 mM (tear)	0–30 mM (blood)	N/A	0–40 mM (blood)	0–40 mM (blood)	0.05–1 mM (tear)
Full scale measurable current	100 µA	40 nA	100pA	1.16 μA	1 µA	150 nA
Temperature Sensor	YES	YES	NO	YES	NO	NO
Power consumption (µW)	<600	50	50 (after rectifier)	250	2	3
EEPROM on-chip	YES	NO	NO	NO	NO	NO
Technology	0.35 µm CMOS	0.13 µm CMOS	0.6 µm CMOS	0.13 µm CMOS	0.18 µm CMOS	0.13 µm CMOS
Chip size (mm^2^)	14.5 (system size)	2.4	1.36	9.98	1.69	0.36
Storage capacitor	On-chip	On-chip	N/A	Off-chip	Off-chip	On-chip
Wirelessly readout distance (cm)	7	3	N/A	N/A	4	15

**Figure A1:**
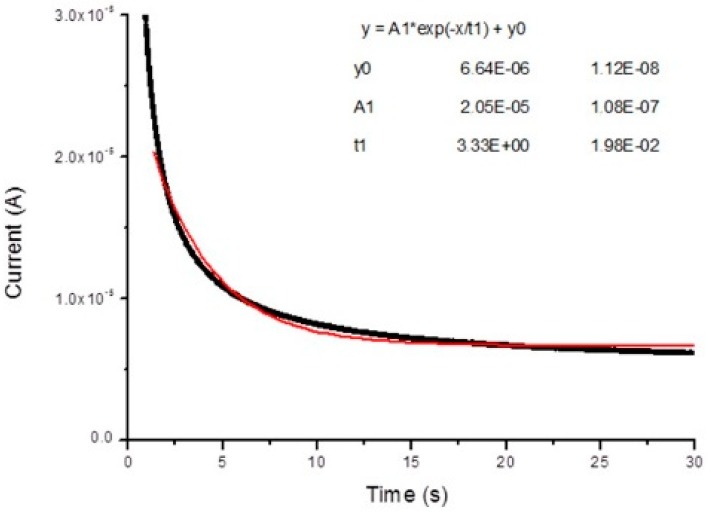
Exponential fit of the experimental points. The measured decay time is 3.3 s and the steady-state current 6.6 µA.

**Figure A2:**
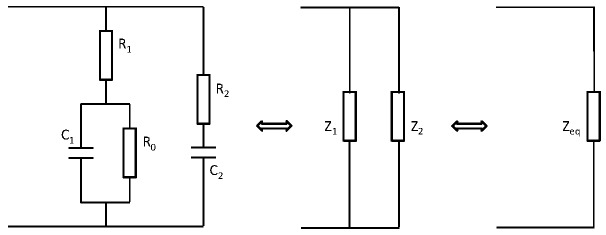
Equivalent electrical circuit.

**Figure A3:**
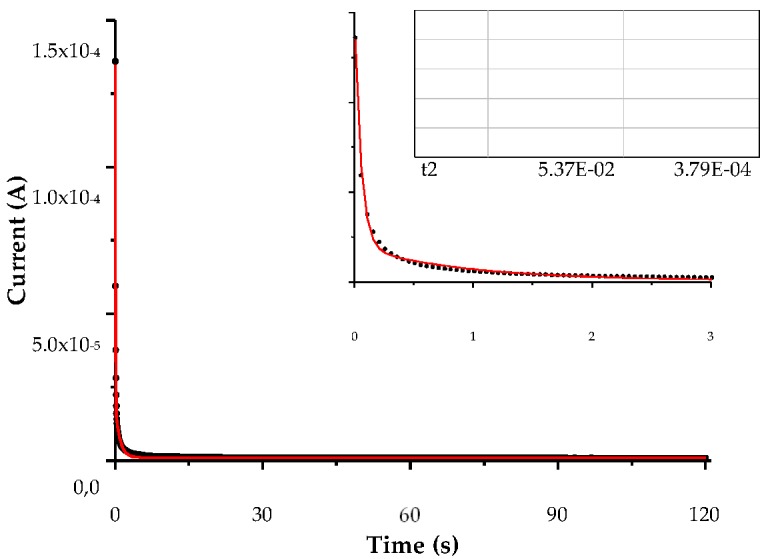
Amperometric measurements. The black dots correspond to the experimental point and the red solid line to the fitting curve. The inset shows a zoom to the beginning of the experiment (first 3 s).

## Appendix A

**Electrical Model of the Sensor [19]**

The current is monitored as a function of time in amperometric measurements when applying a step of voltage of 600 mV using the lab potentiostat (µAutolab Type III, Metrohm). Experiments are carried out with a solution of hydrogen peroxide H_2_O_2_ in PBS (Phosphate Buffer Saline–Sigma Aldrich, Germany) at a concentration of 1 mM.

The steady-state current and the decay time are determined by fitting the experimental points with an exponential function (Figure A1).

The maximum current measured is determined by extracting the maximum value of the measured current from the experimental points. The values of the parameters *i*_0_, *I_M_* and *τ* are presented in Table A1.

**Table A1:** Measurement values of the steady-state current, peak current and decay time.

	Value	Unit
*i*_0_	6.64	µA
*I_M_*	3.82	mA
*τ*	3.33	s

In order to perform simulation for the optimization of the design and layout of the nano-potentiostat circuit an electrical model is needed to imitate the sensor and the electrolyte solution.

A more sophisticate circuit, as displayed in Figure A2, is considered; a resistance and a capacitance in series are additionally coupled in parallel to describe the presence of the electrical double layer on the surface of the sensor.

Experimental data points are recorded by performing amperometric measurements using a solution of H_2_O_2_ at a concentration of 0.1 mM, leading to a steady-state current of roughly 1 µA. The experimental points and the corresponding fit are shown on Figure A3.

The fitting curve perfectly describes the experimental data points, both at very short time and longer time. The considered equivalent circuit allows mimicking realistically the electrochemical cell. The electrical elements can then be derived from the fitting parameters, as stated by the equations established previously. The obtained values are presented in Table A2.

**Table A2:** Calculated values of the electrical elements.

Device	Value	Unit
R0	600	kΩ
R1	30	kΩ
C1	30	µF
R2	4.3	kΩ
C2	125	µF

## References

[1] Liao Y.-T., Yao H., Lingley A., Babak Parviz B., Otis B.P. (2012). A 3-µW CMOS Glucose Sensor for Wireless Contact-Lens Tear Glucose Monitoring. IEEE J. Solid State Circuits.

[2] Leon-Salas W., Kanneganti S., Halmen C. Development of a smart RFID-based corrosion sensor. Proceedings of the 2011 IEEE Sensors.

[3] Boutet P.-A., Manen S. Low power CMOS potentiostat for three electrodes amperometric chemical sensor. Proceedings of the Faible Tension Faible Consommation (FTFC).

[4] Yoon E., Yun K.-S. Development of a wireless environmental sensor system and MEMS-based RF circuit components. Proceedings of the 13th International Conference on Solid-State Sensors, Actuators and Microsystems (TRANSDUCERS ’05).

[5] (2010). ISO/IEC 18000-3:2010 Information technology. Part 3: Parameters for air interface communications at 13.56 MHz. Radio Frequency Identification for Item Management.

[6] Fedtschenko T., Utz A., Stanitzki A., Hennig A., Lüdecke A., Haas N., Kokozinski R. A low-power wireless nano-potentiostat for biomedical applications with ISO 18000-3 interface in 0.35 µm CMOS. Proceedings of the 2018 Twelfth International Conference on Sensing Technology (ICST2018).

[7] Hennig A., Lauko J., Grabmaier A., Wilson C. Wireless Tear Glucose Sensor. Proceedings of the 28th European Conference on Solid-State Transducers (EUROSENSORS 2014).

[8] Ahmadi M.M., Jullien G.A. (2009). A wireless-implantable microsystem for continuous blood glucose monitoring. IEEE Trans. Biomed. Circuits Syst..

[9] Huang W.-J., Liu S.-I. (2008). Capacitor-free low dropout regulators using nested Miller compensation with active resistor and 1-bit programmable capacitor array. IET Circuits Devices Syst..

[10] Kinoshita K. (1992). Electrochemical Oxygen Technology.

[11] Mailand M., Getzlaff S. Transimpedance-amplifier-based subtraction principle for optimum signal resolution in mixed-signal current sensor systems. Proceedings of the 9th International New Circuits and Systems Conference.

[12] Kakerow R.G., Kappert H., Spiegel E., Manoli Y. Low power single chip CMOS poteniostat. Proceedings of the 8th International Conference on Solid-state Sensors and Actuators, and Eurosensors IX.

[13] Ginetti B., Jespers P.G.A., Van de Meulebroecke A. (1992). A CMOS 13-b Cyclic RSD A/D Converter. IEEE J. Solid State Circuits.

[14] Schmidt A., Kappert H., Heiermann W., Kokozinski R. A cyclic RSD analog-digital-converter for application specific high temperature integrated circuits up to 250 °C. Proceedings of the International High Temperature Electronics Conference (HiTEC).

[15] Kownacka A.E., Vegelyte D., Joosse M., Anton N., Toebes J., Lauko J., Buzzacchera I., Lipinska K., Wilson D.A., Geelhoed-Duijvestijn N. (2018). Glucose as an Alternative to Painful Finger-Prick for Diabetes Management Utilizing a Biopolymer Coating. Biomacromolecules.

[16] Xiao Z., Tan X., Chen X., Chen S., Zhang Z., Zhang H., Wang J., Huang Y., Zhang P., Zheng L. (2015). An implantable RFID sensor tag toward continuous glucose monitoring. IEEE J. Biomed. Health Inform..

[17] Guan S., Gu J., Shen Z., Wang J., Huang Y., Mason A. Wireless powered implantable bio-sensor tag system-on-chip for continuous glucose monitoring. Proceedings of the 2011 IEEE Biomedical Circuits and Systems Conference (BioCAS).

[18] Dehennis A.D., Mailand M., Grice D., Getzlaff S., Arthur E., Colvin A.E. A near-field-communication (NFC) enabled wireless fluorimeter for fully implantable biosensing applications. Proceedings of the 2013 IEEE International Solid-State Circuits Conference Digest of Technical Papers.

[19] Pierrat S. (2014). Personal communication.

